# Pre-procedural determination of device size in left atrial appendage occlusion using three-dimensional cardiac computed tomography

**DOI:** 10.1038/s41598-021-03537-9

**Published:** 2021-12-16

**Authors:** Iksung Cho, William D. Kim, Oh Hyun Lee, Min Jae Cha, Jiwon Seo, Chi Young Shim, Hui-Nam Pak, Boyoung Joung, Geu-Ru Hong, Heidi Gransar, Seung Yong Shin, Jung-Sun Kim

**Affiliations:** 1 Division of Cardiology, Severance Cardiovascular Hospital, Yonsei University College of Medicine, Seoul, South Korea; 2 Division of Cardiology, Department of Internal Medicine, Yonsei University College of Medicine and Cardiovascular Center, Yongin Severance Hospital, Yongin, South Korea; 3 Department of Radiology, Chung-Ang University Hospital, Seoul, South Korea; 4 Department of Imaging and Medicine and the Smidt Heart Institute, Cedars-Sinai Medical Center, Los Angeles, CA USA; 5 Division of Cardiology, Chung-Ang University Hospital, 102 Heukseok-ro, Dongjak-gu, Seoul, 03722 South Korea; 6 Division of Cardiology, Department of Internal Medicine, Yonsei University, 50-1, Yonsei-Ro, Seodaemun-gu, Seoul, 03722 South Korea

**Keywords:** Interventional cardiology, X-ray tomography

## Abstract

The complex structure of the left atrial appendage (LAA) brings limitations to the two-dimensional-based LAA occlusion (LAAO) size prediction system using transesophageal echocardiography. The LAA anatomy can be evaluated more precisely using three-dimensional images from cardiac computed tomography (CT); however, there is lack of data regarding which parameter to choose from CT-based images during pre-procedural planning of LAAO. We aimed to assess the accuracy of measurements derived from cardiac CT images for selecting LAAO devices. We retrospectively reviewed 62 patients with Amplatzer Cardiac Plug and Amulet LAAO devices who underwent implantation from 2017 to 2020. The minimal, maximal, average, area-derived, and perimeter-derived diameters of the LAA landing zone were measured using CT-based images. Predicted device sizes using sizing charts were compared with actual successfully implanted device sizes. The mean size of implanted devices was 27.1 ± 3.7 mm. The perimeter-derived diameter predicted device size most accurately (mean error = − 0.8 ± 2.4 mm). All other parameters showed significantly larger error (mean error; minimal diameter = − 4.9 ± 3.3 mm, maximal diameter = 1.0 ± 2.9 mm, average diameter = − 1.6 ± 2.6 mm, area-derived diameter = − 2.0 ± 2.6 mm) than the perimeter-derived diameter (all *p* for difference < 0.05). The error for other parameters were larger in cases with more eccentrically-shaped landing zones, while the perimeter-derived diameter had minor error regardless of eccentricity. When oversizing was used, all parameters showed significant disagreement. The perimeter-derived diameter on cardiac CT images provided the most accurate estimation of LAAO device size regardless of landing zone eccentricity. Oversizing was unnecessary when using cardiac CT to predict an accurate LAAO size.

## Introduction

Atrial fibrillation (AF) is the most common sustained arrhythmia and can lead to several life-threatening complications including ischemic stroke, systemic thromboembolism, and heart failure^1^. The prevention of ischemic stroke in patients with AF is essential, and oral anticoagulation (OAC) is considered the standard treatment^2^. The majority (> 90%) of thrombi develop within the left atrial appendage (LAA) of patients with AF^3^; therefore, percutaneous left atrial appendage occlusion (LAAO) is an alternative non-pharmacological treatment for patients with contraindications to OAC or those who experience recurrent stroke despite OAC treatment^4–6^.

The size and shape of the LAA widely vary among individuals^7^. A deeper understanding of the anatomy of a patient’s LAA is essential before performing LAAO, as faulty device sizing or improper device positioning may result in peri-device leakage or device embolization^3^. The usual pre-procedural evaluation of the LAA is accomplished using two-dimensional trans-esophageal echocardiography (TEE) in order to assess the orifice and landing zone diameter, and to exclude the presence of a thrombus within the appendage^8,9^. Amplatzer Cardiac Plug (ACP) or Amulet devices (Abbott Vascular, Santa Clara, CA, USA) are commonly used for percutaneous LAAO, and the proper device size is determined by measuring the maximum width of the landing zone via TEE^9^. However, the complex structure of the LAA limits the sizing accuracy of two-dimensional imaging, leading to unfavorable outcomes^10^.

Cardiac computed tomography (CT) provides accurate three-dimensional images of the heart and can be electrocardiography (ECG)-gated, which allows for the detailed visualization of cardiac structures during systole and diastole^11^. Recent studies have reported that CT helps improve device sizing and contributes to a more efficient and safer LAAO procedure than the use of TEE to predict the device size^11–14^. However, CT-based pre-procedural planning for LAAO is still under development, as there is still a need for identification of the optimal diameter to use in device sizing in each device type^15^. This study aimed to assess the accuracy of pre-procedural device sizing using three-dimensional cardiac CT, and to identify the parameter that predicts the most accurate LAAO device size.

## Methods

### Patient enrollment

**Figure 1:**
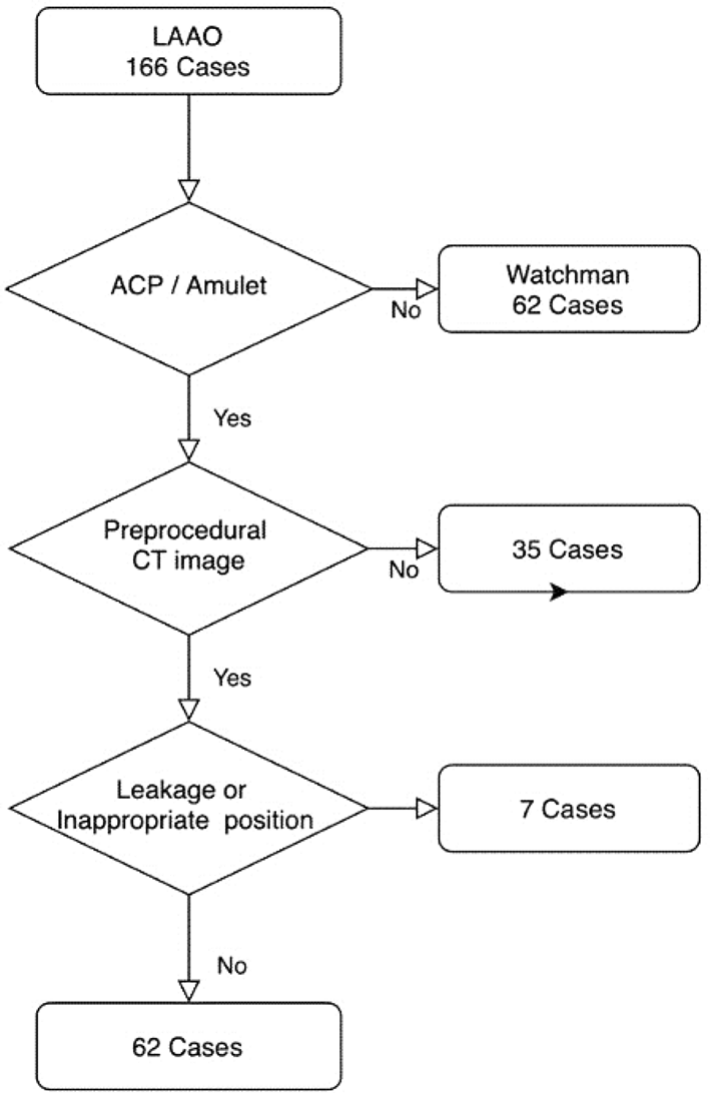
Patient flowchart. *LAAO*: Left ATRIAL appendage occlusion; *ACP*: amplatzer cardiac plug; *CT*: computed tomography.

A total of 166 patients who underwent a percutaneous LAAO device implantation procedure between January 2014 and June 2020 were identified from a Korean multicenter LAAO registry. Patients with a successfully implanted ACP or Amulet device (St. Jude Medical, Minneapolis, MN, USA) (n = 104) were included in the study, whereas patients with Watchman devices implanted were excluded due to the difference in sizing method. Exclusion criteria included the following: (1) patients without pre-procedural cardiac CT (n = 35); and (2) significant peri-device leakage > 3 mm in width, diagnosed on color doppler or an inappropriate position of the device diagnosed on TEE 36 weeks after procedure (n = 7). The final analysis included data from 62 patients who underwent an anatomical and functionally successful LAAO device implantation (Fig. 1). All experiments and methods were performed in accordance with relevant guidelines and regulations. This study was approved by the institutional review board of each institution (Chung-Ang University Hospital, Severance Hospital) and complied with the Declaration of Helsinki. The institutional review board of each institution (Chung-Ang University Hospital, Severance Hospital) waived the requirements of informed consent due to the retrospective nature of this analysis.

### Pre-procedural CT image acquisition

Pre-procedural multi-phasic CT was performed using Philips iCT 256 scanner (Philips Healthcare, Cleveland, OH, USA) (slice collimation = 64 × 0.625, tube voltage = 120 kV, and gantry rotation time = 270 ms) with retrospective ECG-gating and ECG-based tube current modulation. Patients with a heart rate > 65 bpm were administered oral ß-receptor blockers (atenolol 50 mg; Tenormin^®^, AstraZeneca, Sweden) before CT scan is performed. All the patients received 0.8 mg of nitroglycerin sublingually. Using the bolus tracking technique (Bolus Pro Ultra; Philips Healthcare), the contrast-enhanced scanning was initiated after 10 s of triggering with a trigger threshold of 110 Hounsfield Units (HU) ascending aorta. Approximately 50–70 mL of contrast agent (Iomeron 400, 400 mg iodine/mL; Bracco Imaging SpA, Milan, Italy) was injected through the antecubital vein (injection rate = 4.5–5 mL/s) followed by 50 mL of 1:1 mixed contrast saline chaser (4 mL/s) using a dual-head power injector (Stellant; Medrad, Pittsburgh, PA, USA). The presence or absence of an LAA thrombus was assessed using a delayed scan performed 1 min after the contrast injection using prospective ECG-gating centered at 40% of the R-R interval. Images were reconstructed at 0–90% of the R-R interval in 10% increments with a 20-cm field of view, 512 × 512-pixel matrix, 0.9 mm slice thickness, and 0.45-mm image increments with hybrid iterative reconstruction (iDose4; Philips Healthcare) using a medium soft-tissue convolution kernel (XCB, Philips Healthcare, Cleveland, OH, USA).

### CT image analysis and measurement

CT images were saved as Digital Imaging and Communications in Medicine (DICOM) files and imported into a commercially available software package (3mensio Workstation version 10.1, Pie Medical Imaging, Maastricht, The Netherlands). The images were analyzed by experienced imaging cardiologists (Supplement Fig. 1). In detail, the LAA was automatically located, and landmarks were placed at the left circumflex artery and coumadin ridge to locate the LAA ostium. After adjusting the plane angle, the landing zone was set as 10 mm distal to the ostium. The following ostium and landing zone measurements were automatically obtained: the minimal diameter, maximal diameter, average diameter, area-derived diameter, and perimeter-derived diameter. The maximal diameter was defined as the largest distance observed after repeating the measurements between each point. The minimal diameter was the shortest distance that was found in this process, and the average diameter was the mean value of the two. The landing zone area was measured using the shoelace algorithm, summing the divisions of voxels within the area^16,17^. The perimeter was calculated using the length along the lumen line, which is a direct result of lumen segmentation. The area and perimeter-derived diameters were calculated using the equation for the circumference of a circle, dividing each measurement by pi $(\pi )$. Supplement Fig. 2 shows representative cases with eccentric or circular landing zones. The eccentricity index (EI) of the landing zone was calculated for each patient as [1 − (minimal diameter/maximal diameter)], assuming that the shape was similar to an ellipse.

### Device size prediction and evaluation

The device size was predicted using each parameter by choosing the closest lobe size from the observed diameter value. The accuracy of each device size prediction method based on the minimal, maximal, average, area-derived, and perimeter-derived diameters was evaluated by using the size of actually implanted devices as reference. This fulfilled all of the following criteria: (1) optimal position; (2) optimal shape (tire-shape); (3) no leakage on the follow-up TEE; and (4) no thrombus on or adjacent to the LAAO on the follow-up TEE. The mean and mean absolute errors between the predicted size and reference were compared. The mean error was defined as the mean difference between predicted device size and actual device size, and the mean absolute error is the mean value of absolute differences between the two sizes.

To assess the association of eccentricity on the discrepancy in the sizing methods, the error of device size prediction was analyzed in accordance with EI. Previous studies have shown that EI > 0.15 predicts significant residual leak after LAAO procedure, and sizing discrepancy was significant at EI = 0.19^14,18^. We divided the total cases into subgroups (EI > 0.2 and EI ≤ 0.20) and compared the error in device size prediction.

**Figure 2:**
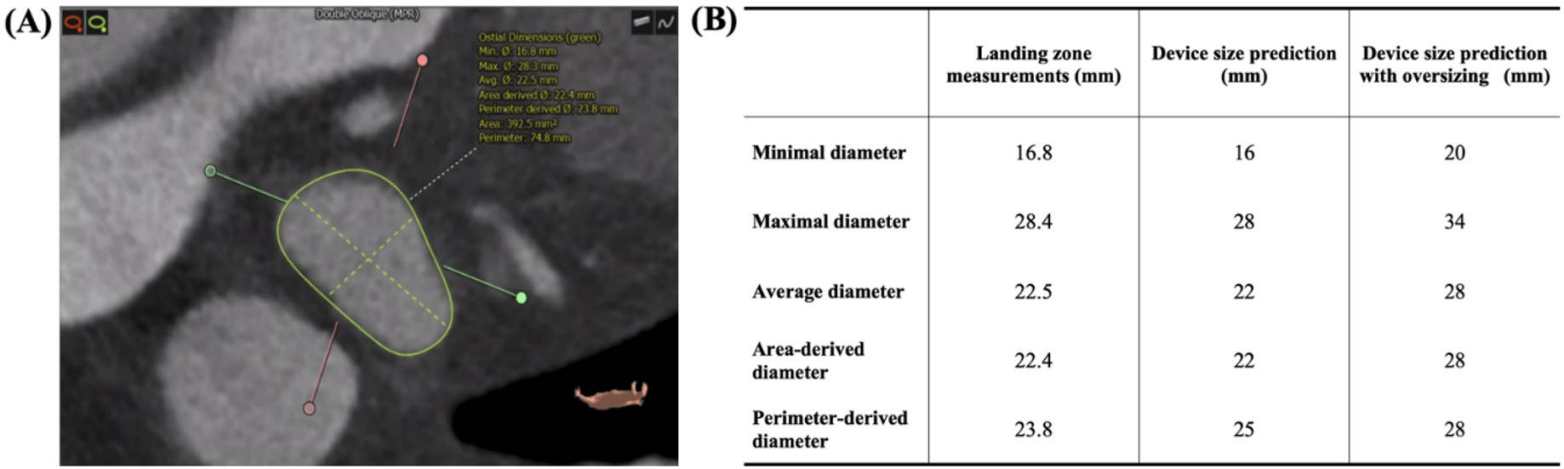
Device size prediction using landing zone measurements. (**A**) The landing zone of the left atrial appendage is located, and five different parameters are acquired: minimal, maximal, average, area-derived, and perimeter-derived diameter. Analysis was performed by 3mensio Workstation version 10.1 (Pie Medical Imaging, Maastricht, The Netherlands). (**B**) Device size is predicted by choosing the closest size to each parameter. Device size prediction with oversizing is performed by choosing the corresponding device size according to sizing charts.

The size prediction method with oversizing used the conventional oversizing system with sizing charts (Fig. 2, Supplement Fig. 3). The mean and mean absolute errors were calculated to evaluate the accuracy of oversizing when CT-based images are used to predict the device size.

### Statistical analyses

The absolute error was tested to verify whether perimeter-derived diameter was statistically different from other measurements, using paired t-test. In addition, the agreement between the device size, predicted using CT measurements, and actual device size was evaluated using Bland–Altman analysis.

Categorical variables are presented as percentages, and continuous variables are presented as means ± standard deviations. All tests were two-sided, and *p*-values < 0.05 were considered statistically significant. All analyses were performed using MedCalc statistical software (version 14.12.0, MedCalc Software Inc., Mariakerke, Belgium) and STATA statistical software (version 14.2, StataCorp LLC, College Station, TX).

## Results

### Baseline characteristics

The baseline characteristics of the 62 patients included in this study are shown in Table 1. The mean patient age was 71 ± 9.8 years, and 39 patients (62.9%) were males. A history of major bleeding incidence or predisposition to bleeding was found in 35.5% of the patients. Nearly 64.5% patients had recurrent stroke episodes despite the use of OAC. Twenty-six patients (41.9%) had a history of stroke, and their mean CHA_2_DS_2-_VASc score was 4.0 ± 1.8. Amulet devices were implanted in 37 patients (59.7%) and ACP devices in 25 (40.3%). The mean lobe size of the implanted devices was 27.1 ± 3.7 mm.

**Table 1:** Patients’ clinical characteristics.

Characteristics	Value
Age	71 ± 9.8
Sex, male	26 (41.9%)
Body mass index	24.1 ± 3.4
Hypertension	26 (83.9%)
Diabetes mellitus	18 (29.0%)
Heart failure	22 (27.7%)
Stroke	26 (41.9%)
Vascular disease	25 (40.3%)
Major/minor bleeding	22 (35.5%)
HAS-BLED score	2.6 ± 1.1
CHA_2_DS_2_VASc score	4.1 ± 1.8
**Indication**	
Prior major bleeding or predisposition to bleeding	22 (35.5%)
Recurrent stroke despite OAC	40 (64.5%)
**Device**	
Amplatzer cardiac plug	25 (40.3%)
Amulet	37 (59.7%)
Implanted device size	27.1 ± 3.7

### Device sizing from landing zone measurements

The parameters of the LAA ostium and landing zone were measured, and the mean values are shown in Table 2. The mean minimal diameter of the landing zone was 22.1 ± 4.5 mm, while the maximal diameter was 29.0 ± 5.0 mm. The perimeter-derived diameter of the landing zone was 26.3 ± 4.3 mm, being slightly larger than average (25.5 ± 4.3 mm) and area-derived diameters (25.3 ± 4.3 mm). The predicted device size was determined for each measured parameter; the minimal diameter gave the smallest size of 22.2 ± 4.3 mm, while the maximal diameter gave the largest size of 28.1 ± 4.3. The perimeter-derived diameter estimated a device size of 26.3 ± 3.9 mm, which was again larger than predicted sizes from average (25.5 ± 4.1 mm) and area-derived diameters (25.0 ± 4.2 mm).

**Table 2:** Diameters and predicted device sizes.

Diameter	Measurements and device size prediction			Prediction accuracy	
	Ostium (mm)	Landing zone (mm)	Predicted device size (mm)	Mean error* (mm)	Mean absolute error^†^ (mm)
Minimal	25.1 ± 5.8	22.1 ± 4.5	22.2 ± 4.3	− 4.9 ± 3.3	5.0 ± 3.1
Maximal	35.6 ± 5.9	29.0 ± 5.0	28.1 ± 3.8	1.0 ± 2.9	2.1 ± 2.2
Average	30.4 ± 5.7	25.5 ± 4.3	25.5 ± 4.1	− 1.6 ± 2.6	2.1 ± 2.2
Area-derived	30.1 ± 5.7	25.3 ± 4.3	25.0 ± 4.2	− 2.0 ± 2.6	2.4 ± 2.3
Perimeter-derived	30.9 ± 5.7	26.3 ± 4.3	26.3 ± 3.9	− 0.8 ± 2.4	1.6 ± 1.9

Data are reported as means ± SDs.

*The mean of the differences between predicted device size and actual device size.

^†^The mean of the absolute differences between predicted device size and actual device size.

### Accuracy of predicted device size compared with actual implanted device

**Figure 3:**
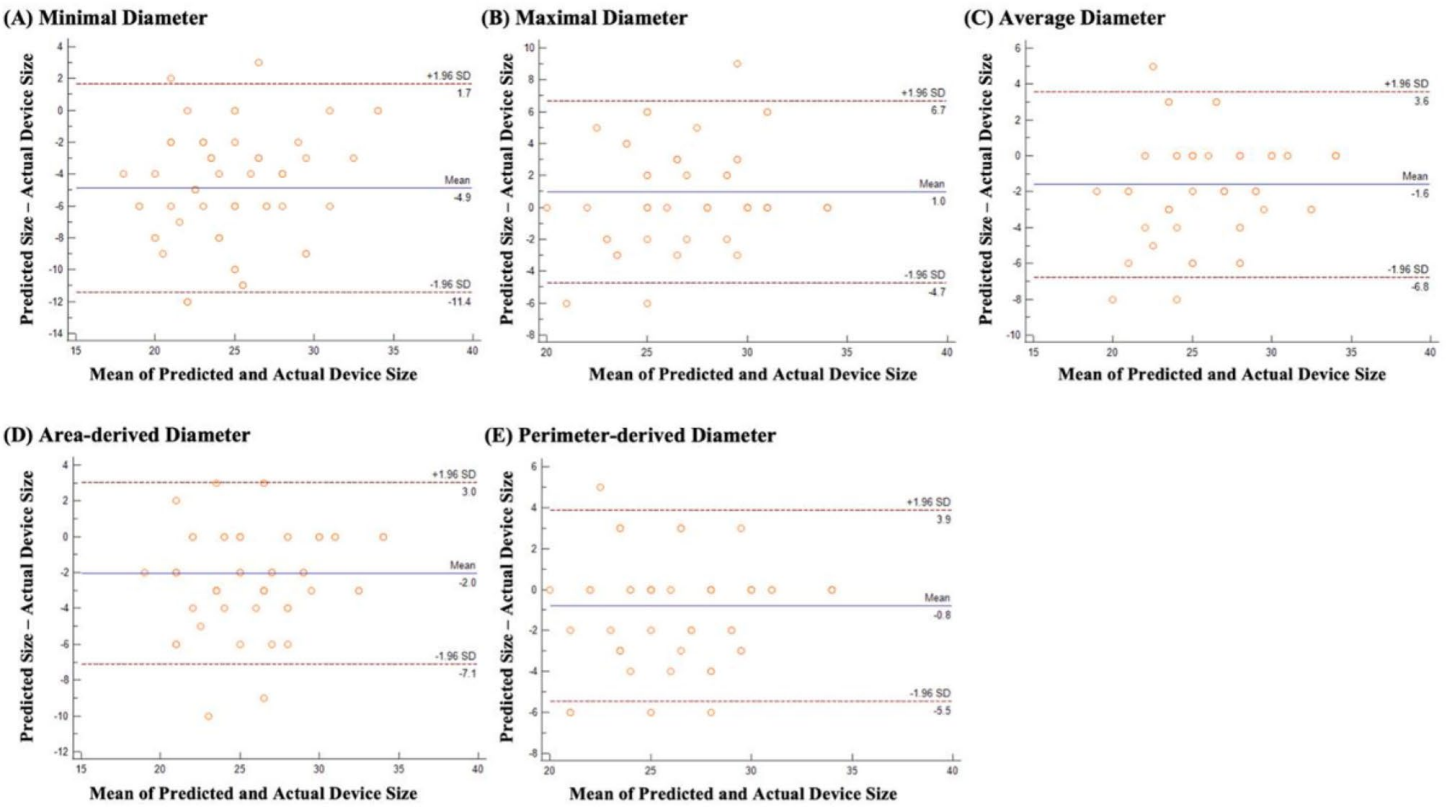
Bland–Altman plot comparing the predicted size using each parameter and actual device size of each parameter.

The accuracy of the device sizing method was evaluated by comparing mean and mean absolute errors between predicted and actual implanted device sizes (Table 2) and also by the Bland–Altman method (Fig. 3). The perimeter-derived diameter showed the highest accuracy in predicting device size with minimal error (mean error = − 0.8 ± 2.4 mm, mean absolute error = 1.6 ± 1.9 mm), while device size predicted from the minimal diameter showed the most significant error (mean error = − 4.9 ± 3.3 mm, mean absolute error = 5.0 ± 3.1 mm). The maximal diameter led to overestimation of device size (mean error = 1.0 ± 2.9), while all other parameters showed underestimated results.

As the perimeter-derived diameter resulted in a minimal error in device sizing, the absolute value of the differences was tested to verify whether it was statistically different from other measurements (Table 3). The predicted device sizes determined using the perimeter-derived diameter were significantly different from the predicted sizes when the minimal (mean error = 3.42 ± 3.25, *p* < 0.001), average (mean error = 0.52 ± 1.36, *p* = 0.004), and area-derived diameters (mean error = 0.77 ± 1.77, *p* = 0.001) were used. The predicted device sizes determined using the perimeter-derived diameter were not significantly different from the predicted sizes determined using maximal diameter (mean error = 0.44 ± 2.51, *p* = 0.177).

**Table 3:** Differences between size recommendations obtained using the perimeter-derived diameter and other diameters.

Variables	Paired differences				P-value
	Mean ± SD	S.E. mean	95% CI		
			Lower	Upper	
Perimeter—minimal	3.42 ± 3.25	0.41	2.59	4.25	< 0.001
Perimeter—maximal	0.44 ± 2.51	0.32	− 0.20	1.07	0.177
Perimeter—average	0.52 ± 1.36	0.17	0.17	0.86	0.004
Perimeter—area	0.77 ± 1.77	0.22	0.33	1.22	0.001

### Eccentricity index and device size selection

**Figure 4:**
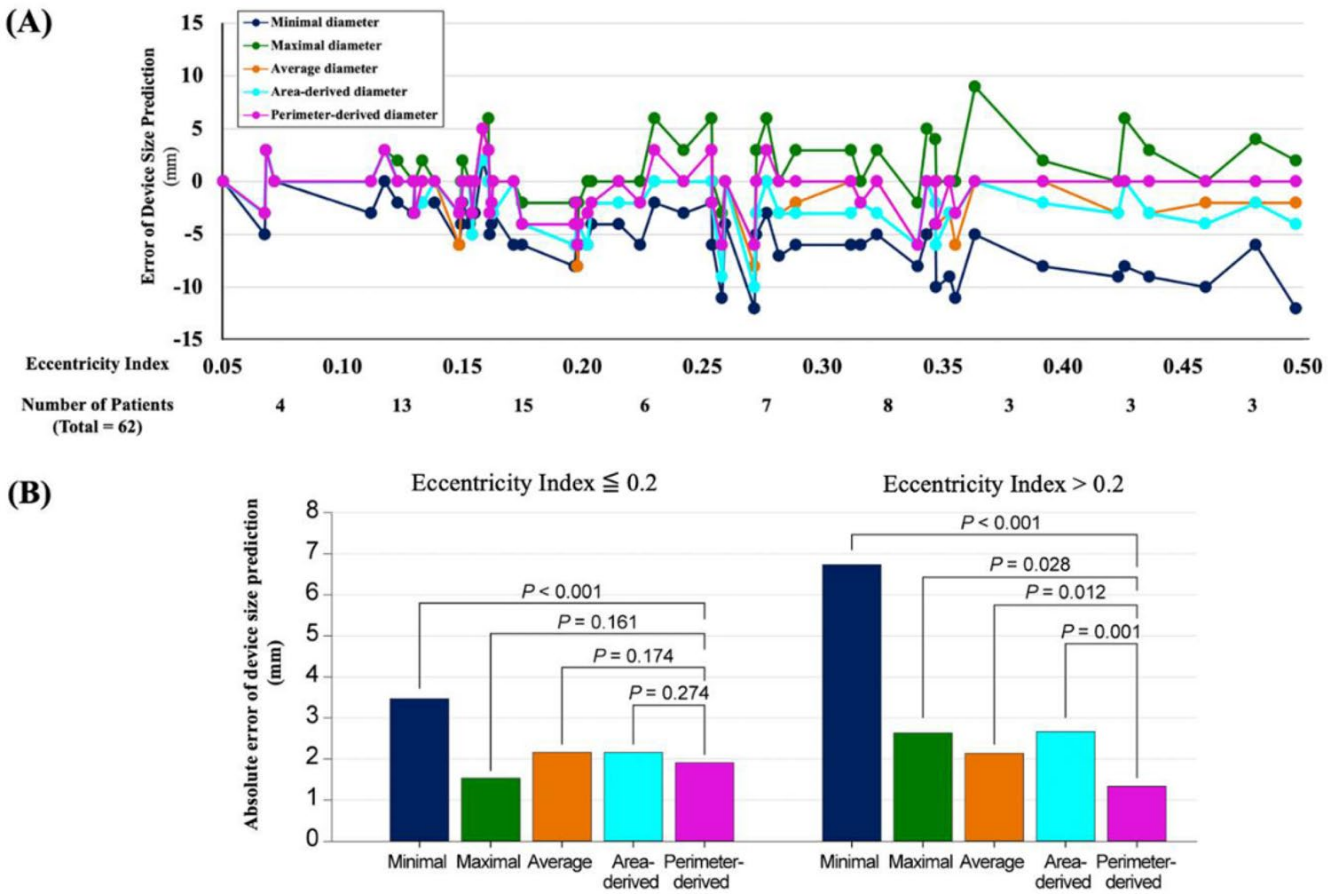
Error of device size prediction according to eccentricity index. (**A**) Error in the predicted device sizes from each parameter according to eccentricity index. (**B**) Absolute error of device sizing from each parameter in eccentric (EI > 0.2) and non-eccentric (EI ≤ 0.2) groups.

The mean EI was 0.23 ± 0.11 (range: 0.05–0.5). Approximately half of the patients (48.4%) had an EI > 0.2, while only 6.5% had an EI < 0.1. The difference between predicted device sizes obtained using each parameter and actual device size according to EI is shown in Fig. 4. When the EI was > 0.2, the perimeter-derived diameter showed the least absolute error in device size prediction, and all other parameters showed significant difference from it (all *p* for difference < 0.05). When the EI was ≤ 0.2, the minimal diameter alone showed significant error, while the other parameters showed no difference. Overall, the perimeter-derived diameter predicted the device size most accurately, regardless of EI.

### Device size prediction with oversizing

The predicted lobe sizes determined using the conventional TEE oversizing method are shown in Table 4 and Supplement Fig. 4. Oversizing resulted in a mean device size of 3.0 ± 1.9 mm larger than the prediction without oversizing. The minimal diameter still underestimated the device size (mean predicted device size = 25.3 ± 4.2 mm; mean error = − 1.8 ± 3.5 mm) with oversizing. All the other parameters overestimated device sizes; the area-derived diameter showed the smallest error but was still significantly large (mean error = 1.4 ± 3.0, mean absolute error = 2.3 ± 2.4).

**Table 4:** Predicted device sizes using oversizing.

Diameter	Measurements and device size prediction			Prediction accuracy	
	Ostium (mm)	Landing zone (mm)	Predicted device size (mm)	Mean error* (mm)	Mean absolute error^†^ (mm)
Minimal	25.1 ± 5.8	22.1 ± 4.5	25.3 ± 4.2	− 1.8 ± 3.5	2.8 ± 2.8
Maximal	35.6 ± 5.9	29.0 ± 5.0	30.7 ± 3.2	3.6 ± 3.6	3.8 ± 3.4
Average	30.4 ± 5.7	25.5 ± 4.3	28.6 ± 3.7	1.5 ± 3.1	2.4 ± 2.5
Area-derived	30.1 ± 5.7	25.3 ± 4.3	28.5 ± 3.5	1.4 ± 3.0	2.3 ± 2.4
Perimeter-derived	30.9 ± 5.7	26.3 ± 4.3	29.2 ± 3.5	2.1 ± 3.1	2.8 ± 2.5

Data are reported as means ± SDs.

*The mean of the differences between the predicted device size and actual device size.

^†^The mean of the absolute differences between the predicted device size and actual device size.

## Discussion

In this multicenter study, using three-dimensional cardiac CT image-based measurements to predict the size of LAAO devices, the perimeter-derived diameter of the landing zone was found to be the most accurate predictor of device size. The perimeter-derived diameter was advantageous when the cross-sectional shape of the LAA was eccentric (EI > 0.2). Additionally, oversizing based on the TEE sizing chart was unnecessary due to the superior spatial resolution of CT imaging.

### LAAO planning with CT measurements

TEE is a conventional pre-procedural method for evaluating the LAA. It is used to measure the width of the ostium and landing zone in multiple planes; the maximal diameter of the landing zone is used to determine the ACP or Amulet device size^8^. However, CT images have higher spatial resolution than TEE images and can be reconstructed into three-dimensional images, thereby providing the operator with a better understanding of the morphology of the LAA and its surrounding structures^11,13,15^. Therefore, CT is being used increasingly for the pre-procedural assessment of other percutaneous procedures, including transcatheter aortic valve replacement^19^. Recent studies have reported that CT provides better accuracy for LAAO planning than TEE^13,20–22^, whereas it is still unclear which parameter should be used for sizing when using CT measurements to plan LAAO^15^. Therefore, our study aimed to establish a practical method of successfully using CT-based parameters to predict LAAO device sizes for ACP and Amulet.

### Perimeter-derived diameter

The ACP and Amulet devices both have eight different sizes in 2–3 mm increments. In this study, the mean difference of the perimeter-derived diameter and actual device width was significantly smaller than 2 mm (− 0.8 ± 2.4 mm), indicating a minimal error and excellent match. Previous studies have suggested that the perimeter is the most dependable parameter for evaluating the LAA ostium. An expert recommendation by Korsholm et al. suggested the need for identification of the optimal use of the perimeter-derived diameter in different devices^15^. Wang et al. compared the different parameters during different cardiac phases and found that the perimeter-derived diameter had minimal changes (1–2 mm) and was reliable for reproducing the ostium^23^. However, this study compared the parameters measured using two-dimensional oblique and three-dimensional measurement methods, focusing on the reproducibility of the LAA ostium rather than the accuracy of the sizing. In our study, we used successfully implanted devices as the reference to compare predicted device sizes based on CT measurements and were able to present the superior accuracy of the perimeter-derived diameter compared with other parameters. More recently, Jia et al. compared the parameters measured using three-dimensional printed models and found a good correlation between the perimeter of the LAA orifice and LAmbre™ device size^24^. However, the ability of 3D printing to reflect actual cardiac anatomy may be limited, as the volume within the chambers changes throughout the cardiac cycle. Thus, our findings were consistent with previous studies that suggested the perimeter-derived diameter as the most accurate parameter in reproducing the LAA ostium, while further showing the optimal use of it in CT-based LAAO planning with ACP and Amulet devices.

### Underlying mechanism of superior accuracy in sizing with perimeter-derived diameter

In the current study, the minimal, average, and area-derived diameters underestimated the size of the LAAO device. Significant undersizing may lead to complications, including device malpositioning, embolization, or peri-device leakage. A comparison of the parameters in this study is shown in Supplement Fig. 5. The average diameter was calculated as the arithmetic mean of the minimal and maximal diameters ($\frac{D1+D2}{2}$). When the minimal diameter was significantly smaller than the maximal diameter, or when the shape was more eccentric, the average diameter was relatively a small value. The area-derived diameter of an ellipse can be calculated as the geometric mean of the minimal and maximal diameters ($\sqrt{D1*D2})$. Inequality of the two mean values indicates that the geometric mean is always less than or equal to the arithmetic mean, leading to an underestimation of size when using area-derived diameters.

The EI was identified as the important factor when comparing the results of device size for each parameter. The cross-sectional shape of the LAA ostium and landing zone is typically elliptical or irregular, while the occluding device is circular^25–27^. This difference may lead to a discrepancy between the predicted sizes and actual device sizes. EI can be used to determine the shape of the LAA ostium and landing zone, as the shape is more circular when the EI is approximately 0. When the occluding devices are inserted, the shape of the landing zone deforms to adapt to the device^23^. This adaptation does not lead to significant changes in the LAA parameters in patients with more circular EI. However, in patients with more eccentric landing zones, the adaptation significantly changes the minimal, maximal, average, and area-derived diameters, while the perimeter-derived diameter does not change significantly. In our study, the discrepancy between the predicted device size was greater when the EI was > 0.2. Almost half of the patients in this study (48.4%) had an EI > 0.2, indicating that using diameters other than the perimeter-derived diameter may lead to a mismatch in the device selection.

The maximal diameter measured in this study was similar to the actual implanted device size and not significantly different from the perimeter-derived diameter. The LAA is a relatively distensible structure within the heart, serving as a volume reservoir during the systolic phase^28^. This anatomical characteristic may allow for the maximal diameter to remain as an important measurement in LAAO, along with the perimeter-derived diameter. However, the insertion of grossly large devices may lead to malpositioning of the device and post-procedural complications, including device embolization, peri-device leakage, thrombus formation, and cardiac tamponade^18,29^. Additionally, in more eccentric cases with EI > 0.2, the maximal diameter showed significant error compared with the perimeter-derived diameter, leading to overestimation of device size. Therefore, in patients with highly eccentric LAA ostium shapes, the perimeter-derived diameter may be the most accurate parameter for device size selection.

### Unnecessity of oversizing with CT-based measurements

Our study also assessed the need for oversizing when planning for LAAO using CT. Sizing charts provided by the device manufacturer were used to determine the ideal device size correlating to the obtained measurements^6^. These charts typically suggest oversized device disc diameters due to an underestimation of the dimensions when two-dimensional TEE is used^30^. Three-dimensional TEE has improved the accuracy for the assessment of the true LAA orifice compared with two-dimensional TEE^31^; however, the measurements were smaller than those obtained using cardiac CT^30^.

When the oversizing method was used with CT-based measurements, each parameter was significantly mismatched with the actual device size. Oversizing improved the accuracy of the predicted device size when the minimal diameter was used from a mean error of − 4.8 ± 3.3 mm to − 1.8 ± 3.5 mm. However, the minimal diameter underestimates the lobe size irrespective of oversizing, and this improvement is clinically irrelevant as the minimal diameter is not used independently for the sizing of LAAO devices. All other parameters significantly overestimated the device size when oversizing was used. Therefore, oversizing may be unnecessary when CT images are used for pre-procedural LAAO planning.

## Limitations

This study has a few limitations. First, only patients who underwent LAAO with no complications or device size mismatching were included, and our results were not directly compared with those obtained using the conventional TEE method. However, we used the actual implanted devices as reference to compare the device sizes predicted using different parameters. Second, this was a retrospective study and may have been influenced by unobserved confounders and selection or referral biases. Thus, the clinical feasibility and usefulness of this sizing method, such as the improved success rate, decreased procedure time, and decreased number of attempts, must be verified in future prospective studies. Lastly, this study only included patients implanted with ACP or Amulet devices, which led to the exclusion of a significant number of patients with Watchman devices. This exclusion criterion was based on the different sizing techniques used for Watchman devices. As the use of perimeter-derived diameter without oversizing may be considered for other devices including the Watchman device, further studies regarding the use of parameters from CT-based images for the pre-procedural planning of other LAAO devices may help establish optimal guidelines for each device.

## Conclusion

When using cardiac CT-based measurements for LAAO planning, the perimeter-derived diameter is the most accurate parameter to predict the device size. The eccentricity of the LAA ostium is a critical factor responsible for the discrepancy among the CT-based measurements, and the perimeter-derived diameter is advantageous in patients with a highly eccentric LAA ostium. Future prospective studies should consider evaluating the clinical utility of the perimeter-based size determination method.

## Supplementary Information

Supplementary Figures.

## Data Availability

The datasets generated during and/or analysed during the current study are available from the corresponding author on reasonable request.
