# Supplementary material for: Pre-procedural determination of device size in left atrial appendage occlusion using three-dimensional cardiac computed tomography
Source: Sci Rep. 2021 Dec 16;11:24107. doi: 10.1038/s41598-021-03537-9 (PMC8677741; doi:10.1038/s41598-021-03537-9)
Supplement: Supplementary file 1 — Supplementary Figures. [file 41598_2021_3537_MOESM1_ESM.docx]

Supplement Figure 1. Cardiac computed tomography image analysis using 3mensio Workstation version 10.1 (Pie Medical Imaging, Maastricht, The Netherlands)


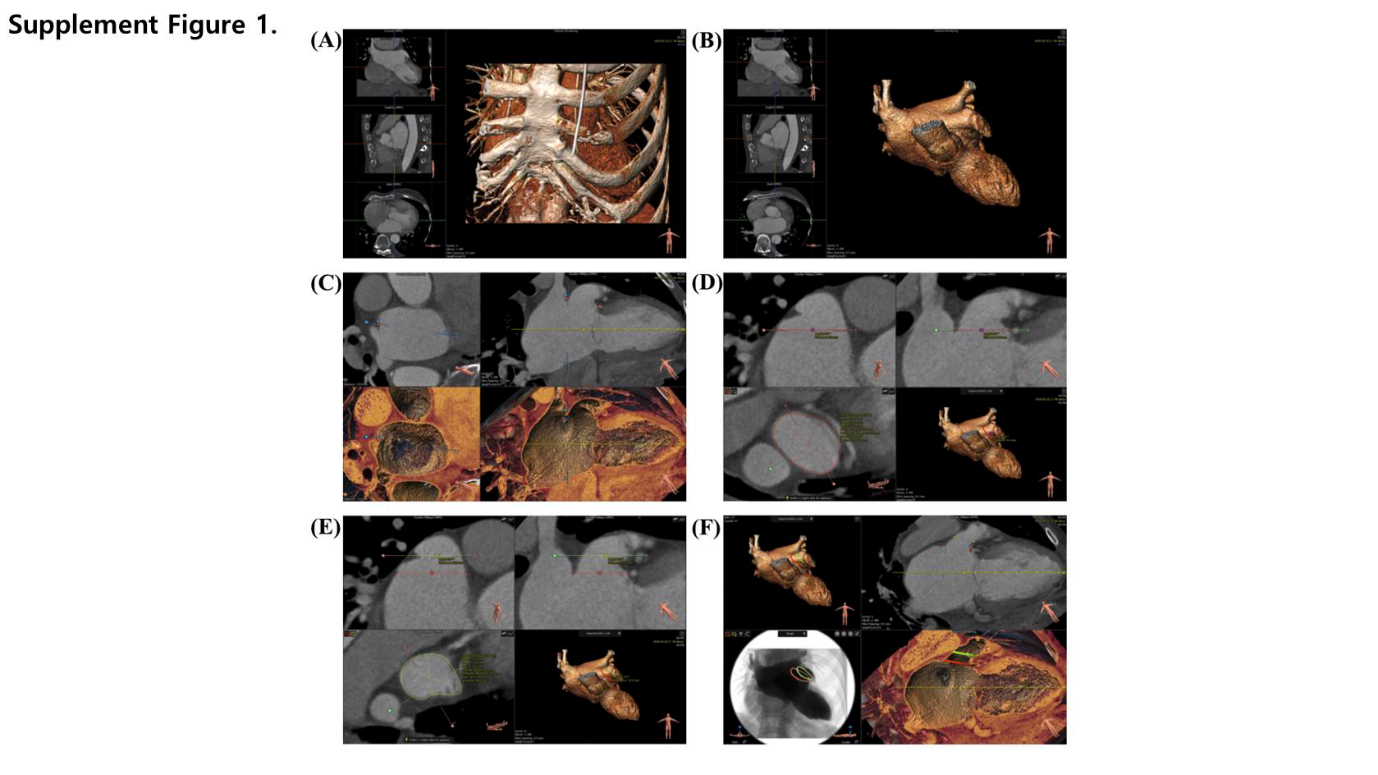
 (A) Cardiac computed tomography images are imported, and (B) the left atrial appendage (LAA) is automatically located. (C) Landmarks are placed at the coumadin ridge and left circumflex artery. (D) The LAA ostium is located and measured. (E) The landing zone is defined as the area 10 mm distal to the ostium and is measured. (F) Virtual images of the ostium and landing zone are available in a three-dimensional model, CT image, and fluoroscopy.

Supplement Figure 2. Representative cases of landing zone measurements


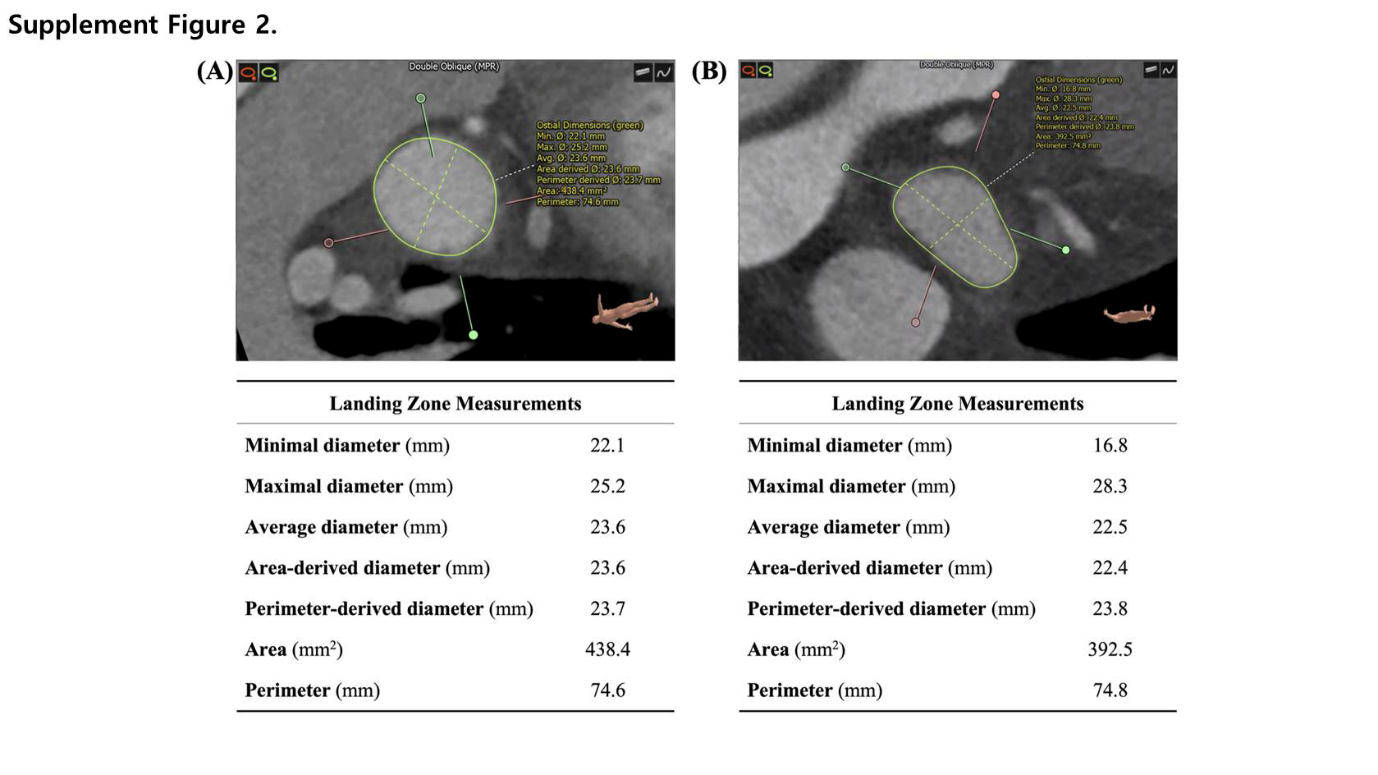
(A) A circular-shaped landing zone with less difference between parameters

(B) An eccentric landing zone with a significant difference between parameters

Analysis was performed by 3mensio Workstation version 10.1 (Pie Medical Imaging, Maastricht, The Netherlands).

Supplement Figure 3. Sizing charts for Amulet and Amplatzer cardiac plug


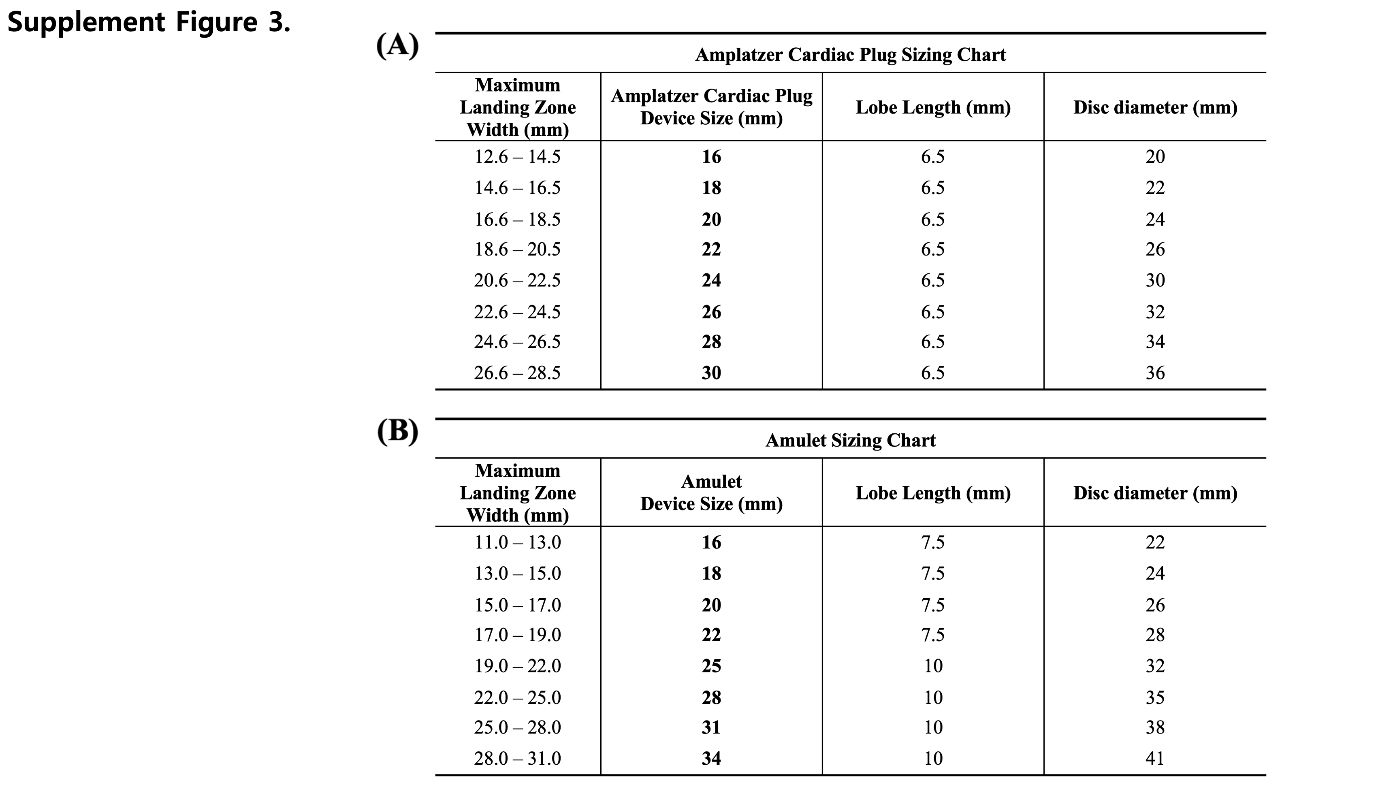


(A) Sizing chart for Amplatzer cardiac plug

(B) Sizing chart for Amulet

Supplement Figure 4. Bland-Altman plots comparing the predicted size using each parameter with oversizing and the actual device size


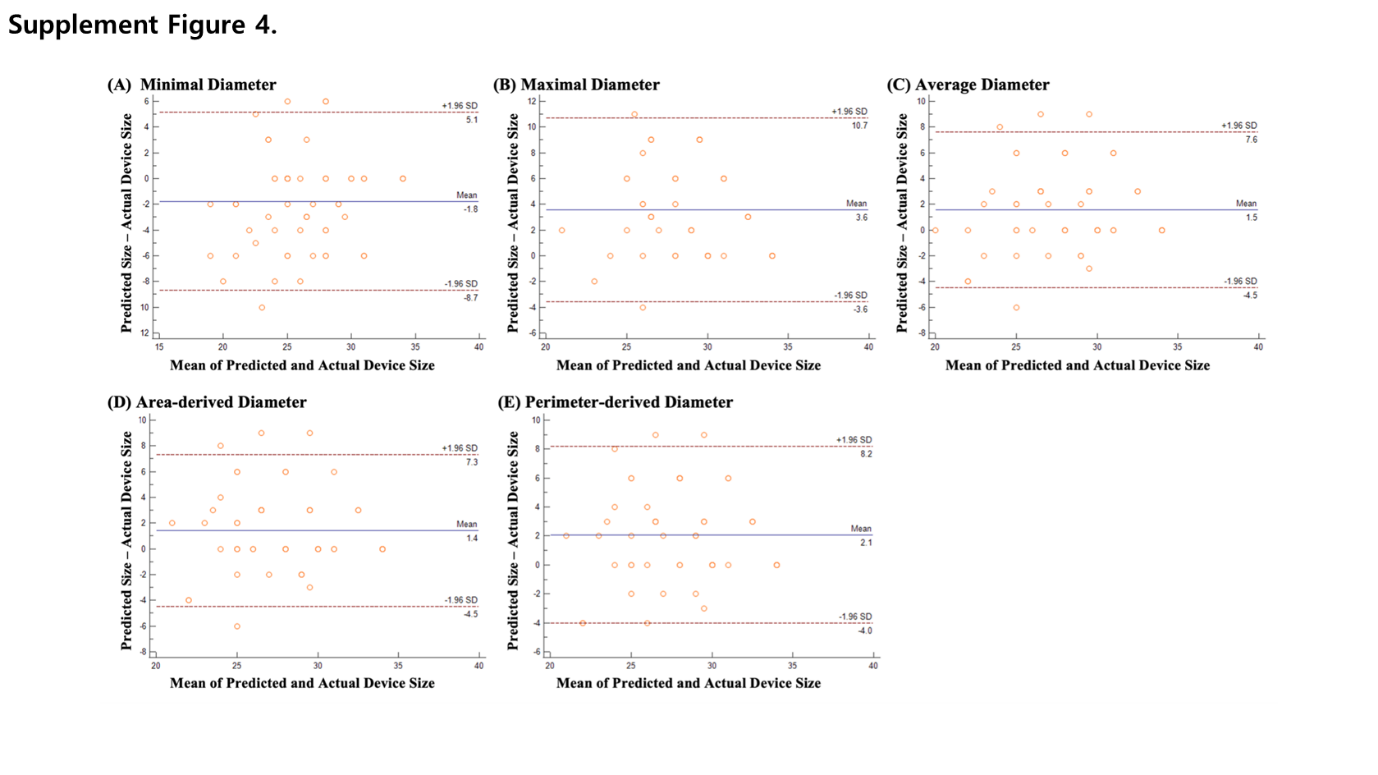


Supplement Figure 5. Diameters of an oval


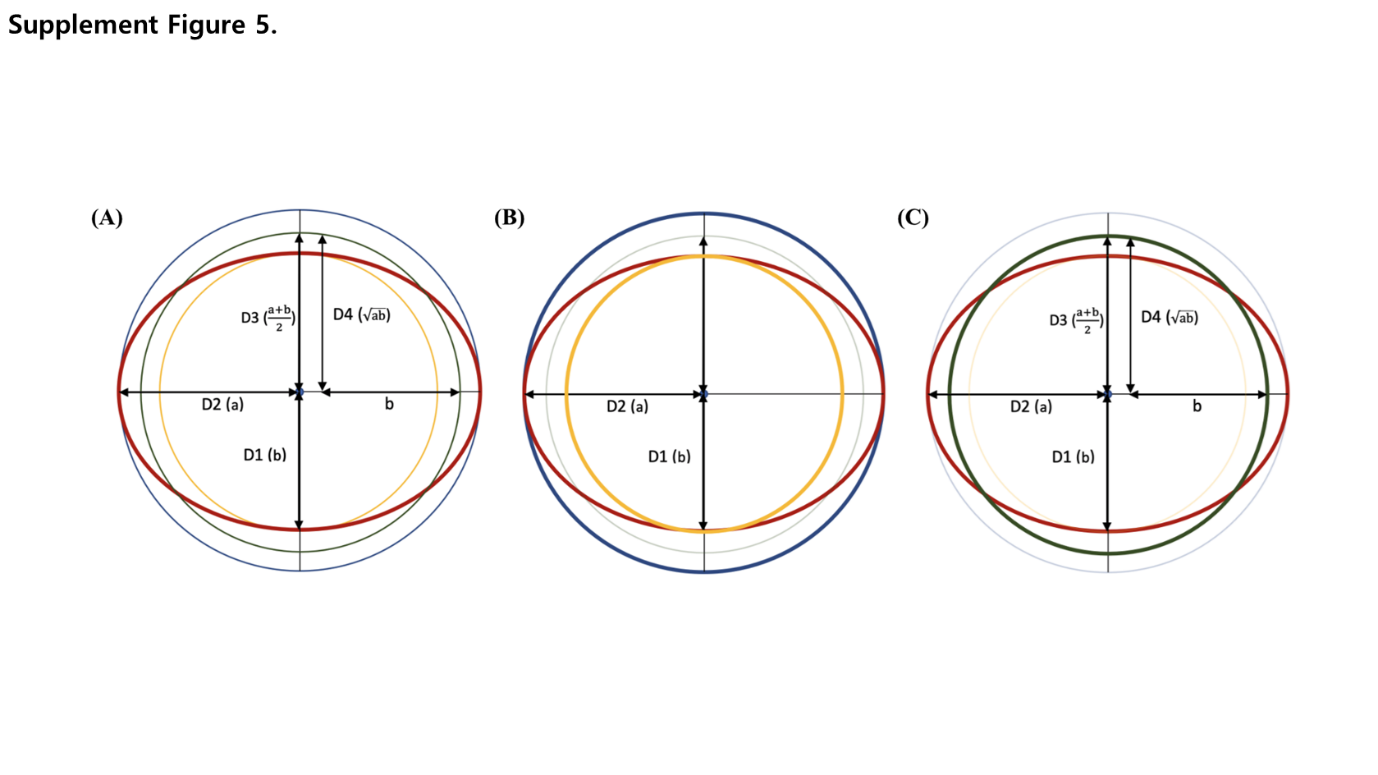


(A) The red line represents an oval with a long radius (a) and a short radius (b).

(B) The blue and yellow lines represent a circle with a radius of (a) and (b), respectively.

(C) The green line represents a circle with a radius of the arithmetic mean of (a) and (b). The geometric mean of the two radiuses is expressed, showing a smaller value than the arithmetic mean.
